# Dosimetric evaluation of incidental irradiation to the axilla during whole breast radiotherapy for patients with left-sided early breast cancer in the IMRT era

**DOI:** 10.1097/MD.0000000000004036

**Published:** 2016-07-01

**Authors:** Jayoung Lee, Shin-Wook Kim, Seok Hyun Son

**Affiliations:** a Department of Radiation Oncology, Pusan National University Yangsan Hospital, Yangsan, Republic of Korea; b Department of Radiation Oncology, Incheon St. Mary's Hospital, College of Medicine, The Catholic University of Korea, Seoul, Republic of Korea.

**Keywords:** early breast cancer, incidental irradiation to the axilla, intensity-modulated radiotherapy, whole breast radiotherapy

## Abstract

The purpose of this study was to compare the dosimetric parameters for incidental irradiation to the axilla during whole breast radiotherapy (WBRT) with 3-dimensional conformal radiotherapy (3D-CRT) and intensity-modulated radiotherapy (IMRT). Twenty left breast cancer patients treated with WBRT after breast-conserving surgery (BCS) were enrolled in this study. Remnant breast tissue, 3 levels of the axilla, heart, and lung were delineated. We used 2 different radiotherapy methods: 3D-CRT with field-in-field technique and 7-field fixed-beam IMRT. The target coverage of IMRT was significantly better than that of 3D-CRT (*D*_mean_: 49.72 ± 0.64 Gy vs 50.24 ± 0.66 Gy, *P* < 0.001; *V*_45_: 93.19 ± 1.40% vs 98.59 ± 0.30%, *P* < 0.001; *V*_47.5_: 86.43 ± 2.72% vs 95.00 ± 0.02%, *P* < 0.001, for 3D-CRT and IMRT, respectively). In the IMRT plan, a lower dose was delivered to a wider region of the heart and lung. Significantly lower axillary irradiation was shown throughout each level of axilla by IMRT compared to 3D-CRT (*D*_mean_ for level I: 42.58 ± 5.31 Gy vs 14.49 ± 6.91 Gy, *P* < 0.001; *D*_mean_ for level II: 26.25 ± 10.43 Gy vs 3.41 ± 3.11 Gy, *P* < 0.001; *D*_mean_ for level III: 6.26 ± 4.69 Gy vs 1.16 ± 0.51 Gy, *P* < 0.001; *D*_mean_ for total axilla: 33.9 ± 6.89 Gy vs 9.96 ± 5.21 Gy, *P* < 0.001, for 3D-CRT and IMRT, respectively). In conclusion, the incidental dose delivered to the axilla was significantly lower for IMRT compared to 3D-CRT. Therefore, IMRT, which only includes the breast parenchyma, should be cautiously used in patients with limited positive sentinel lymph nodes and who do not undergo complete axillary lymph node dissection.

## 1 Introduction

Since the introduction of breast-conserving surgery (BCS) for early breast cancer, complete axillary lymph node dissection (cALND) has been acknowledged as the treatment of choice for the patients with clinically negative axilla and positive sentinel lymph node biopsy (SLNB) results. However, the literature has failed to show improved clinical outcomes of cALND in the aspect of locoregional control and overall survival.^[1,2]^ Therefore, the American College of Surgeons Oncology Group (ACOSOG) Z0011 phase III trial, in which patients with positive sentinel lymph nodes (SLNs) were randomized to cALND or no further axillary surgery, was started. Both arms received whole breast radiotherapy (WBRT) in the course of treatment. Beyond general awareness, the 2 arms did not show a significant difference in locoregional recurrence rates statistically.^[3]^ This result led to focusing on incidentally irradiated dose to the axilla during WBRT, which can eradicate additional hidden metastasis of axillary lymph nodes. This concept is even more important for early breast cancer patients with 1 or 2 positive SLNs who decide to omit cALND.

Recently, a new radiation technique, intensity-modulated radiotherapy (IMRT), has been increasingly used for WBRT to reduce radiation dose to the adjacent normal organs, especially the heart, in patients with left-sided breast cancer.^[4–6]^ This can also reduce the radiation dose to certain levels of the axilla. Therefore, in this study, we compared the differences in 3-dimensional conformal radiotherapy (3D-CRT) and IMRT according to incidentally irradiated dose to the axilla.

## 2 Materials and methods

### 2.1 Patients

Computed tomography (CT) scans of 20 patients with left-sided early breast cancer treated with radiotherapy (RT) after BCS from 2014 to 2015 were selected for this study. All the patients received WBRT using the 3D-CRT technique, and additional IMRT plans were performed for this comparative study following institutional review board approval (IRB of Incheon St. Mary's Hospital, College of Medicine, the Catholic University of Korea, Reference number: OC15RISI0104).

### 2.2 Simulation and delineation of target volume and organs at risk

For the simulations, patients underwent a CT scan with LightSpeed RT16 CT scanner (General Electric Company, Waukesha, WI). They were immobilized on a breast-tilting board with both arms in the up position with a vac-lock immobilization device. The boundaries of the remaining breast tissue were wired by nonmetallic thread through palpation and visual inspection. The CT images were acquired in 2.5-mm thickness. Eclipse version 8.9 (Varian Medical System, Palo Alto, CA) was used to process CT images, contour the regions of interest, and perform 2 kinds of dose calculations.

The clinical target volume (CTV) of the left breast (remaining breast parenchyma), levels I, II, and III of the axilla, heart, lung, and spinal cord were delineated. For the consistency and reliability of target volume and organs at risk (OARs), all of these were contoured by 1 experienced radiation oncologist and were based on the Radiation Therapy Oncology Group (RTOG) atlas.^[7]^ The breast CTV was edited according to the wired area and some specific CT finding such as a surgical clip and seroma, and we trimmed the anterior border by 3 mm from the skin for skin-sparing treatment planning.

### 2.3 Radiation treatment planning for the IMRT and 3D-CRT

For the IMRT plan, we performed a skin-sparing IMRT.^[8]^ Almost all breast cancer patients suffer from radiation-induced dermatitis during or after the end of RT, and latent severe skin sequelae could occur if not properly treated. Because too small of a distance between breast CTV and skin could result in unnecessarily high dose of radiation to the skin, we separated distance between the breast CTV and skin in the contouring phase mentioned above. Fixed-beam IMRT with 7-field was used for IMRT. Although the angles of each field could vary individually, the intervals were the same in all patients; angle intervals were 45, 30, 20, 20, 30, and 45° in a clockwise direction. The 6 MV photon beam was used for each field, and an analytical anisotropic algorithm (version 8.9.17) was used for dose calculation. The calculation grid was 2.5 × 2.5 mm. The prescribed dose was 50 Gy in 25 fractions, and the plans were optimized to deliver at least 95% of the prescribed dose to 95% of the breast CTV for all 20 patients enrolled in this study. In addition, we tried to reduce the radiation dose to the heart and lung as much as possible while maintaining dose coverage for breast CTV.

For the 3D-CRT plan, to remove unexpected hot spots and improve homogeneities for breast CTV, we used the field-in-field (FIF) technique in addition to the conventional parallel-opposite tangential fields technique (2 oblique beams with an interval of 180°) that is traditionally used for breast cancer patients. Usually, 2 to 4 additional subfields were added. The photon beam energy, algorithm for dose calculation, and grid size used were the same as those of the IMRT plan. The upper margin of the main field was the upper most point, either 0.5 cm above the sternal notch or 2 cm above the breast CTV, and the lower margin was 2 cm below the breast CTV. For valid comparison of dose distribution between the 2 kinds of plans, we normalized D_50_ (the minimum dose for 50% of the target volume) of breast CTV in the 3D-CRT plan to that in the IMRT plan.

### 2.4 Dosimetric parameters and statistical analysis

For comparison of IMRT and 3D-CRT plan, *D*_mean_ (mean dose) and *V*_n_ (percentage of volume receiving more than at least n Gy) were used as dosimetric parameters of each structure. The Wilcoxon signed-rank test were used for comparison of the 2 plans. Statistical analysis was performed using R version 3.2.1 (R Development Core Team, Vienna, Austria) and a *P* value of < 0.05 was considered significant.

## 3 Results

### 3.1 Comparison of dosimetric parameters between the IMRT and 3D-CRT plans

**Figure 1:**
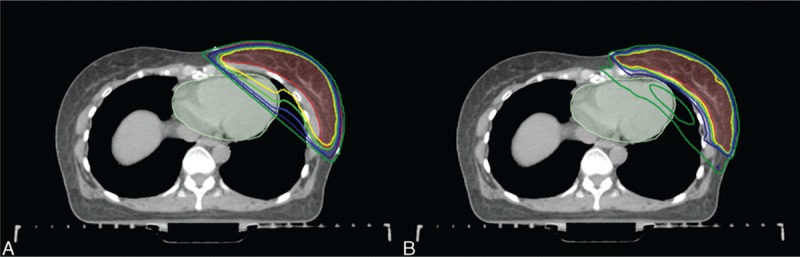
Dose distributions of (A) the 3D-CRT and (B) IMRT plans (breast CTV = semi-lucent red area; heart = semi-lucent pale green area; yellow line = 50 Gy; yellowish green line = 47.5 Gy; blue line = 45 Gy; navy line = 40 Gy; green line = 25 Gy). 3D-CRT = 3-dimensional conformal radiotherapy, CTV = clinical target volume, IMRT = intensity-modulated radiotherapy.

The mean breast CTV was 453.9 ± 156.4 cm^3^, and the mean volumes of axillary levels I, II, III, and total axilla were 28.3 ± 6.8 cm^3^, 14.11 ± 3.83 cm^3^, 5.7 ± 1.5 cm^3^, and 48.8 ± 9.5 cm^3^, respectively. Representative axial images of both plans including target volume and dose distributions are shown in Fig. 1. When compared to the 3D-CRT plan, the IMRT plan had a more conformal dose distribution, similar to the shape of breast CTV. However, the low-dose area in the IMRT plan was much wider than that in the 3D-CRT plan.

The dose coverage for breast CTV was significantly improved by the IMRT plan. The *D*_mean_ of 3D-CRT plan and IMRT plans was 49.72 ± 0.64 Gy and 50.24 ± 0.66 Gy, respectively, which was statistically significant (*P* < 0.001). *V*_45_ and *V*_47.5_ were also more adequate in the IMRT plan than in the 3D-CRT plan (*V*_45_: 93.19 ± 1.40% vs 98.59 ± 0.30%, *P* < 0.001; *V*_47.5_: 86.43 ± 2.72% vs 95.00 ± 0.02%, *P* < 0.001, for 3D-CRT and IMRT, respectively).

**Figure 2:**
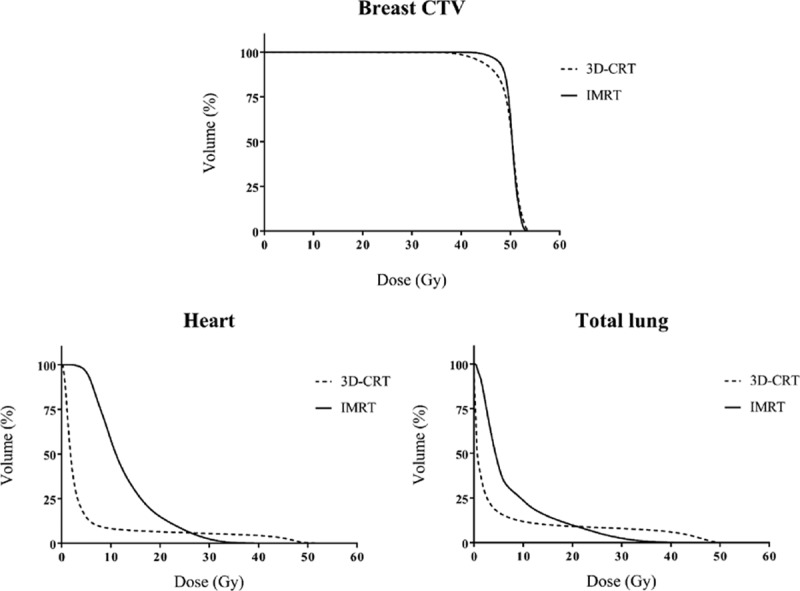
Comparison of dose–volume histograms for breast CTV, heart, and total lung between the 3D-CRT and IMRT plans. 3D-CRT = 3-dimensional conformal radiotherapy, CTV = clinical target volume, IMRT = intensity-modulated radiotherapy.

However, the dose delivered to the OARs such as the heart and lung showed ambivalent features. The mean dose and parameters indicating low-dose irradiation were inferior in the IMRT plan compared to the 3D-CRT plan. However, the parameters indicating high-dose irradiation in the IMRT plan were superior to those in the 3D-CRT plan. In the case of the heart, the *D*_mean_ of the 3D-CRT and IMRT plans was 4.87 ± 1.64 Gy and 12.85 ± 1.55 Gy, respectively, which represents a statistically significant difference (*P* < 0.001). However, *V*_30_ and *V*_40_ were much higher in the 3D-CRT plan than in the IMRT plan (*V*_30_: 5.53 ± 3.07% vs 2.42 ± 1.89%, *P* < 0.001; *V*_40_: 4.38 ± 2.62% vs 0.05 ± 0.10%, *P* < 0.001, for 3D-CRT and IMRT, respectively). In the case of total lung, the *D*_mean_ of the 3D-CRT and IMRT plans was 4.98 ± 1.17 Gy and 7.48 ± 0.66 Gy, respectively, which represents a statistically significant difference (*P* < 0.001). *V*_5_ was also higher in the IMRT plan than in the 3D-CRT plan (*V*_5_: 17.07 ± 4.93% vs 43.04 ± 3.91%, *P* < 0.001, for 3D-CRT and IMRT, respectively). However, *V*_30_ was much higher in the 3D-CRT plan than in the IMRT plan (*V*_30_: 7.97 ± 3.64% vs 2.50 ± 1.13%, *P* < 0.001, for 3D-CRT and IMRT, respectively). We summarized the results in Table 1, and the dose–volume histogram (DVH) of breast CTV, heart, and left lung in the IMRT and 3D-CRT plans is shown in Fig. 2. All values presented in the table satisfied the dose constraint guidelines of the Quantitative Analyses of Normal Tissue Effects in the Clinic,^[9,10]^ and these indicate that both plans are acceptable for treatment.

**Table 1:**
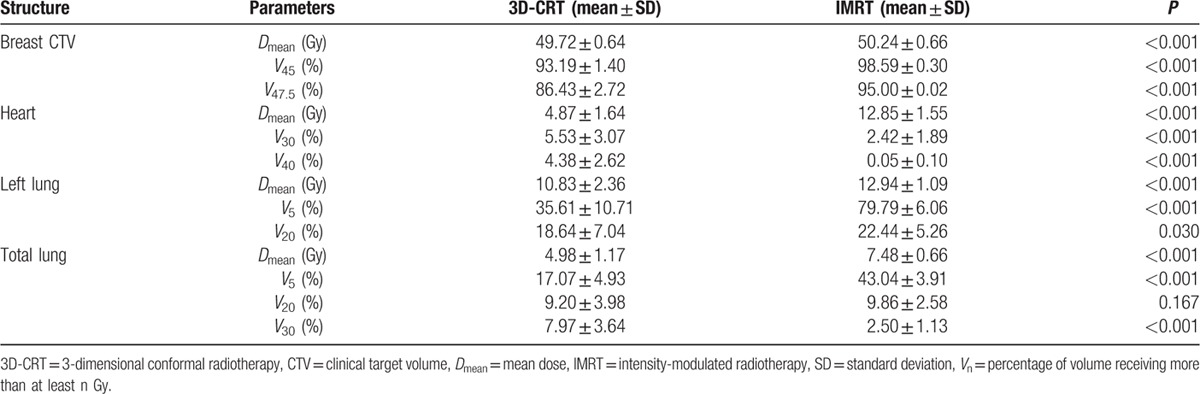
Comparison of dosimetric parameters for breast CTV and organs at risk in the 3D-CRT and IMRT plans.

### 3.2 Incidental axillary dose coverage

**Figure 3:**
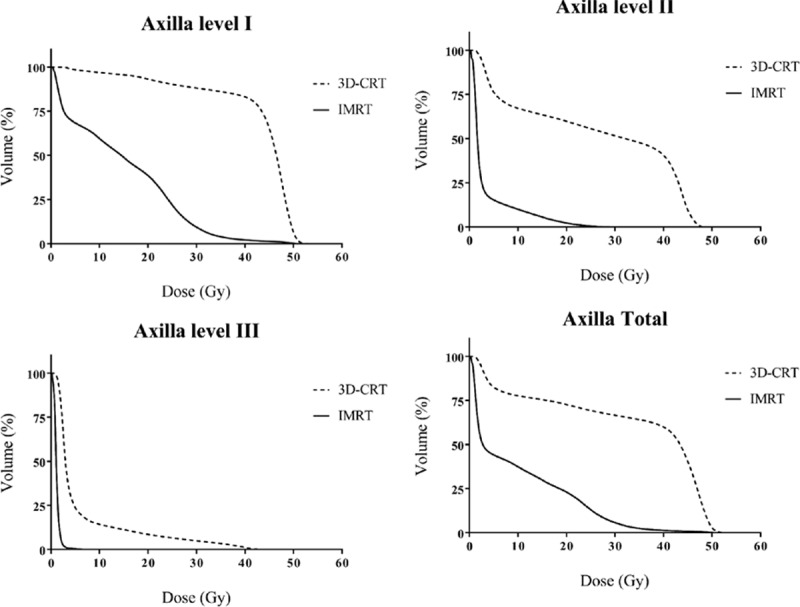
Comparison of dose–volume histograms for each level of the axilla between the 3D-CRT and IMRT plans. 3D-CRT = 3-dimensional conformal radiotherapy, IMRT = intensity-modulated radiotherapy.

The dose delivered to the axilla was significantly lower in the IMRT plan than in the 3D-CRT plan. The *D*_mean_ was 42.58 ± 5.31 Gy vs 14.49 ± 6.91 Gy for level I, 26.25 ± 10.43 Gy vs 3.41 ± 3.11 Gy for level II, 6.26 ± 4.69 Gy vs 1.16 ± 0.51 Gy for level III, and 33.33 ± 6.61 Gy vs 9.89 ± 4.98 Gy for total axilla, for 3D-CRT and IMRT, respectively (*P* < 0.001 for each). Except for the axilla level III, parameters such as *V*_25_, *V*_40_, and *V*_45_ also indicated that the dose delivered to the axilla was lower in the IMRT plan than in the 3D-CRT plan. The detailed values of each parameter for the axilla levels I, II, and III are shown in Table 2, and DVH for each level of the axilla is also shown in Fig. 3.

**Table 2:**
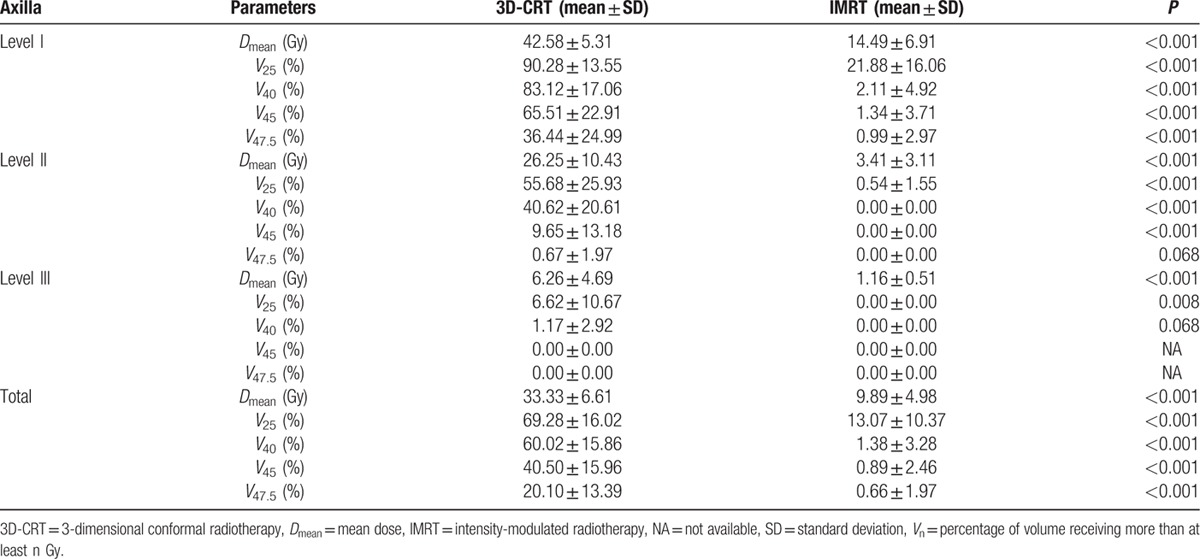
Comparison of dosimetric parameters for each level of the axilla in the 3D-CRT and IMRT plans.

Not surprisingly, this result was thoroughly expected because the improved conformity of IMRT means that less irradiation will be delivered outside of the target volume. In the 3D-CRT plan, because conventional parallel-opposed tangential fields were mainly used, the lower axilla tended to be incidentally irradiated with a higher dose and wider area.

## 4 Discussion

The importance of breast irradiation as a standard procedure in breast-conserving therapy is undeniable for local control. However, incidental axillary irradiation accompanying unexpected axillary control remains controversial. There have been several reports on axillary coverage by conventional radiation with parallel-opposed tangential fields.^[11–14]^ Those reports concluded that the mean dose and percent volume of the receiving >95% of prescribed dose were not sufficiently high. By adjusting the cranial border of the field to just below the humeral head, axillary coverage can be improved.^[14]^ However, the risk of lymphedema of the ipsilateral arm is also increased.^[15]^

As more and more patients with breast cancer are treated with IMRT, a few articles have reported the incidental axillary irradiation by IMRT, even though the technique is relatively simple. Kataria et al analyzed incidental irradiation to the axilla with 3 different radiation techniques: intensity-modulated tangents, 3-dimensional tangents (FIF technique), and standard tangents. They concluded that the lower axilla (level I and II) received a substantial incidental dose with all 3 types of tangent; however, conformal techniques delivered a significantly lower incidental dose to the axilla than the standard tangents.^[16]^ Zhang et al reported dose coverage of the axilla with simplified IMRT (s-IMRT) and for-IMRT (FIF technique with 2 tangential fields) in early breast cancer patients and compared the 2 plans. They concluded that the s-IMRT plan delivered a lower dose to the axilla, and thus caution should be exercised for the centers using the s-IMRT technique.^[17]^

The dosimetric reviews of each level of axilla from the 2 published articles mentioned above and from this study are summarized in Table 3. Although there is a limitation in the direct comparison of different studies and data sets, some differences between the 3 studies could be found. In case of axillary coverage by 3D-CRT, the study of Kataria et al,^[16]^ and this study showed relatively similar mean dose and values of *V*_40,_*V*_45_, and *V*_47.5_ in the lower axilla area (level I and II). However, the values for the same parameters reported by Zhang et al^[17]^ were too low compared to those of Kataria et al and this study. This difference might be due to differences in patient position, contouring extent of the axilla, field extent, and/or IMRT optimization. Also, this finding indicated that even if 3D-CRT is used for WBRT, the dose delivered to the axilla could vary considerably. In the case of IMRT, this study presented noteworthy differences in comparison to the 2 other studies. For all levels of the axilla, a significantly lower radiation dose was delivered.

**Table 3:**
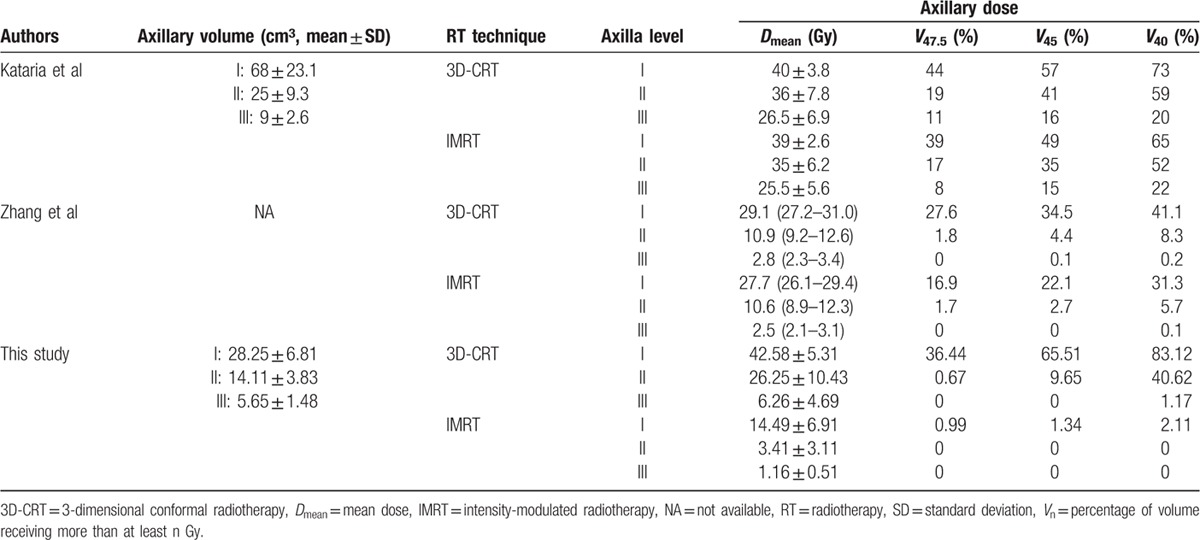
Summary of axillary doses between the 3D-CRT and IMRT plans from previously published studies and this study.

However, the mean dose to each level of the axilla in this study showed large differences between 3D-CRT and IMRT (level I: 42.58 Gy vs 14.49 Gy, level II: 26.25 Gy vs 3.41 Gy, level III: 6.26 Gy vs 1.16 Gy) compared to doses in the Kataria et al study (level I: 40 Gy vs 39 Gy, level II: 36 Gy vs 35 Gy, level III: 26.5 Gy vs 25.5 Gy) and Zhang et al study (level I: 29.1 Gy vs 27.7 Gy, level II: 10.9 Gy vs 10.6 Gy, level III: 2.8 Gy vs 2.5 Gy). In the case of the upper part of axilla (level III), this study showed a significantly lower dose with the IMRT plan. These differences could be due to the degree of IMRT optimization. Because the irradiated dose outside the target volume can vary with the degree of IMRT optimization, the axillary dose can also vary. In addition, the height of the cranial border in 3D-CRT might influence the level of difference between 3D-CRT and IMRT, especially for upper axilla. The cranial boarder of the 3D-CRT in our study was similar to, or slightly higher, from that seen in the IMRT. In the study by Kataria et al, the cranial border of the 3D-CRT was a superior extent of PTV, which means that the cranial border of 3D-CRT might be slightly lower than that of the IMRT. In the study by Zhang et al, the cranial border for 3D-CRT was not described and therefore, could not be compared with that used in our study.

To date, we have not considered the dose delivered to the axilla during IMRT for early breast cancer compared to the cases of advanced disease where the axilla was included in the target volume. However, this carries the potential risk of missing opportunity for regional control of occult metastasis of the axilla, especially for patients with limited positive sentinel lymph nodes who do not undergo cALND. Thus, tailored RT for individual patients might be needed.

## 5 Conclusion

After the ACOSOG Z0011 trial, many clinicians treating breast cancer agreed to omit cALND for patients who meet certain eligibility criteria. They also focused on the incidentally irradiated dose to the axilla by WBRT. However, the majority of the reported studies until that time used standard parallel-opposite tangential fields for WBRT. Thus, we should carefully consider the axilla during IMRT planning procedures for patients with early breast cancer according to the expected risk of axillary lymph node metastasis.
